# Structural, mechanical, dielectric properties and magnetic interactions in Dy^3+^-substituted Co–Cu–Zn nanoferrites

**DOI:** 10.1039/d0ra05274d

**Published:** 2020-07-27

**Authors:** R. H. Kadam, R. B. Borade, M. L. Mane, D. R. Mane, K. M. Batoo, Sagar E. Shirsath

**Affiliations:** Materials Science Research Laboratory Shrikrishna Mahavidyalaya, Gunjoti Osmanabad M.S. India ram111612@yahoo.co.in; Department of Physics, MSS's Arts, Science and Commerce College Ambad Jalna M.S. India; Department of Physics, R. G. Shinde College Paranda Osmanabad M.S. India; Department of Higher Education Maharashtra State, Central Building Pune M.S. India; King Abdullah Institute for Nanotechnology, King Saud University P.O. Box. 2455 Riyadh-1145 Saudi Arabia; Department of Physics, Vivekanand College Aurangabad 431001 M.S. India shirsathsagar@hotmail.com; School of Materials Science and Engineering, University of New South Wales Sydney NSW 2052 Australia

## Abstract

Sol–gel-synthesized Co–Cu–Zn ferrite nanoparticles diluted with Dy^3+^ ions were investigated in terms of their structural, morphological, elastic, magnetic and dielectric properties. X-ray diffraction patterns showed the formation of a single-phase cubic spinel structure. As the concentration of Dy^3+^ ions was increased, the lattice length gradually increased from 8.340 to 8.545 Å, obeying Vegard's law. The Williamson–Hall (W–H) method was employed to observe the change in the lattice strain. Crystallite size obtained from W–H plots followed a pattern similar to that observed using the Scherrer equation. The cation distribution suggested a strong preference of Dy^3+^ ions for the octahedral B site while Cu^2+^ and Fe^3+^ ions were distributed over both A and B sites. The microstructures of the samples were visualized using transmission electron microscopy. Mechanical properties such as stiffness constant, longitudinal and transverse wave velocities, Young's modulus, bulk modulus, rigidity modulus, Poisson's ratio and Debye temperature were investigated by acquiring infrared spectra recorded in the range of 300 to 800 cm^−1^. Replacement of Fe^3+^ ions with the strongly magnetic Dy^3+^ ions increased the saturation magnetization and coercivity. Dielectric constant increased with Dy^3+^ substitution but decreased with applied frequency.

## Abstract 2

Improvement in various properties of Co–Cu–Zn ferrite upon Dy substitution.

## Graphical abstract

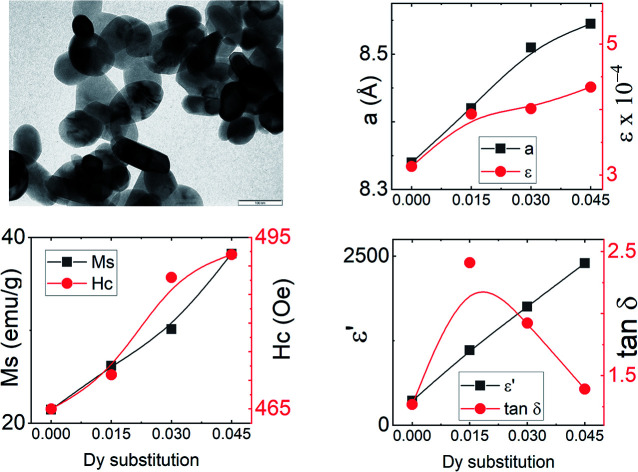

## Introduction

Nanocrystalline spinel oxides such as ferrites display integral properties of magnetization and electrical insulation simultaneously and thus have attracted enormous attention of researchers and have prompted them to intensively investigate these systems. Ferrites offer numerous technological applications including telecommunications and electronic engineering, microwave-absorbing devices, transformer cores, magnetic recording media, magnetic resonance imaging, sensors, computer memory chips, ferro-fluids, radio frequency coil fabrication, catalysis, photocatalysis, magnetically guided drug delivery, hyperthermia, *etc.*^1–6^ Nanodimension spinel ferrites constitute a primary class of nanomaterials having advantageous ferromagnetic properties that have promoted their technological applicability.^7–9^ Among the ferrimagnetic spinel ferrites, CoFe_2_O_4_ with an MgAl_2_O_4_ inverse spinel-like crystal structure displays particularly excellent magnetic properties such as excellent saturation magnetization (*M*_s_ ∼ 80 emu g^−1^), coercivity (*H*_c_ ∼ 3−5 kOe), first-order magnetocrystalline anisotropy constant (*K*_1_ ∼ 10 × 10^6^ erg cm^−1^) and Curie temperature (*T*_c_ ∼ 870 K).^10,11^ Structural, elastic, magnetic and electrical properties of CoFe_2_O_4_ have been reportedly enhanced by the substitution of Cu and/or Zn ions.^1,12,13^

Apparently, properties of spinel ferrite have been shown to be modified by substitutions of rare earth (RE^3+^) ions at the octahedral B site.^14^ These modifications have been attributed to the RE^3+^ ions having large magnetic moments, very large magnetostriction and large magnetocrystalline anisotropy as a result of their strong spin–orbit coupling of the angular momentum and unpaired 4f electrons.

Furthermore, the 4f shell of RE^3+^ ions is covered by 5s^2^5p^6^ electrons and is not influenced by the potential field of the neighbouring metal ions. The substitution of RE^3+^ into ferrites can establish the 4f–3d couplings that govern magnetocrystalline anisotropy and hence improve the magnetic properties of the ferrites.^15,16^ Also, RE^3+^ oxides have good electrical resistivity of >10^5^ Ω cm at room temperature.^17,18^ Among the RE^3+^ ions, substitution of Dy^3+^ ions have yielded particularly marked improvements in the properties of ferrites.^19,20^ In the current work, the structural, elastic, magnetic and dielectric properties of Co_0.4_Cu_0.1_Zn_0.5_Dy_*x*_Fe_2−*x*_O_4_ (where, *x* = 0.0, 0.015, 0.03, 0.045) nanoparticles synthesized using the sol–gel auto-combustion route were revealed, for the first time to the best of our knowledge.

The sol–gel auto-combustion method was used in the present work for the synthesis of Co_0.4_Cu_0.1_Zn_0.5_Dy_*x*_Fe_2−*x*_O_4_ materials since use of this method has been shown to produce nanoparticles. Importantly, relative to other available processes such as co-precipitation and hydrothermal methods, the sol–gel auto-combustion method displays advantages such as the ability to form a desired phase and composition, control over crystal size and aggregation state, short processing time, easy process, and use of inexpensive raw materials.^1^

The main objective of the present work was to investigate the effect of Dy^3+^ ions on the structural, optical and magnetic properties of the Co–Cu–Zn ferrite. Regarding structural properties, the crystallite size and strain effect were investigated systematically. Elastic behavior was investigated using infrared spectroscopy. Magnetic properties such as saturation magnetization and coercivity of the investigated samples were also studied for their possible applications in technological devices.

## Materials and methods

Pure, ultrafine nanopowders of Co_0.4_Cu_0.1_Zn_0.5_Dy_*x*_Fe_2−*x*_O_4_ (*x* = 0.0, 0.015, 0.03, 0.045) were fabricated by applying the sol–gel autocombustion method. Ultrapure ((>99%) metal nitrates of Co(NO_3_)_2_·6H_2_O, Cu(NO_3_)_2_·6H_2_O, Zn(NO_3_)_2_·6H_2_O, Fe(NO_3_)_3_·9H_2_O, and Dy(NO_3_)_3_·9H_2_O) were used in combination with (C_6_H_8_O_7_·H_2_O) as a chelating agent in the molar ratio of 1 : 3. Liquid ammonia was slowly introduced into the solution in order to adjust the pH to be ≅7. Until a viscous gel formed, the whole mixture was continuously stirred at a constant temperature of 90 °C. After a self-ignition process was carried out, the burnt powders were sintered at 700 °C for 6 h and ground in order to obtain the desired fine nanoparticles.

Powder samples were characterized by subjecting them to X-ray diffraction analysis using a Rigaku (*Miniflux II*) diffractometer spanning diffraction angles from 20−80° at a slow rate of 2° per minute together with using Cu-K_α_ radiation (*λ* = 1.5406 Å) at 40 kV. The microstructures and surface morphologies of the samples were investigated using transmission electron microscopy (JEOL JEM 2100). Elasticity parameters were computed from FTIR spectra recorded in the range 300–800 cm^−1^. FTIR measurements were performed on disc-shaped samples made by using ferrite-KBr with a ratio of 1 : 10. Magnetic characterization was carried out using a vibrating sample magnetometer and applying a magnetic field of up to 5 kOe. The dielectric behavior of the samples was investigated by first forming silver-pasted discs of them and then analyzing the discs by using a Hioki HiTESTER 3532-50.

## Results and discussion

### Structural analysis

**Fig. 1:**
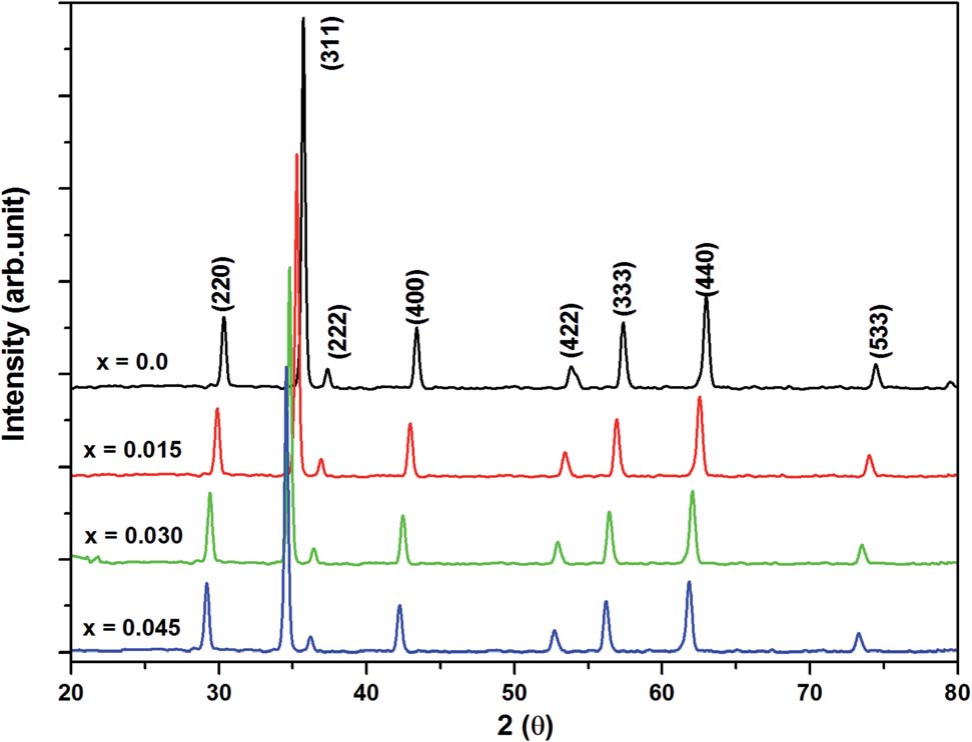
X-ray powder diffraction patterns of all of the investigated samples of Co_0.4_Cu_0.1_Zn_0.5_Dy_*x*_Fe_2−*x*_O_4_.

**Fig. 2:**
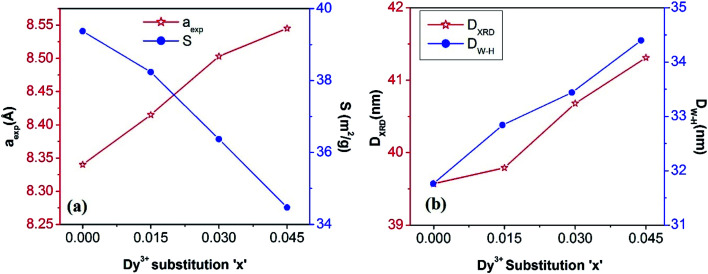
(a) Experimental lattice length (*a*_exp_) and specific surface area (*s*) values and (b) crystallite sizes obtained from XRD (*D*_XRD_) and from W–H plots (*D*_W–H_) of Co_0.4_Cu_0.1_Zn_0.5_Dy_*x*_Fe_2−*x*_O_4_ ferrites.

Bragg reflections from the (220), (311), (222), (400), (422), (333), (440), and (533) planes (indexed by ICCD # 00-008-0234) were observed in the X-ray diffraction (XRD) patterns of Co_0.4_Cu_0.1_Zn_0.5_Dy_*x*_Fe_2−*x*_O_4_ (Fig. 1). These patterns clearly indicated the formation of a single phase, in particular a cubic spinel structure belonging to the space group *Fd*3̄*m*.^23^ Neither any impurity nor a secondary phase was indicated from an analysis of the XRD patterns. Addition of Dy^3+^ ions shifted the peak positions of Co_0.4_Cu_0.1_Zn_0.5_Fe_2_O_4_ slightly towards the lower angles, as depicted in Fig. 1. The experimental lattice length *a*_exp_ was computed by employing the equation^21^
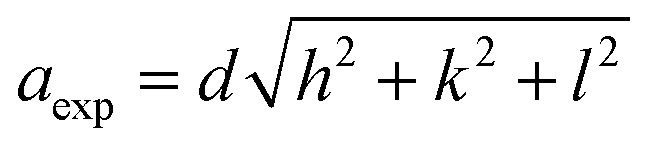
, where (*h k l*) are the Miller indices and *d* is the interplanar distance. As shown in Fig. 2a, the lattice length of Co_0.4_Cu_0.1_Zn_0.5_Dy_*x*_Fe_2−*x*_O_4_ increased from 8.340 to 8.545 Å with the substitution of Dy^3+^ ions. This change in *a*_exp_ can be understood in terms of the ionic radii of the constituent ions, where Fe^3+^ ions of smaller ionic radii (0.67 Å) were replaced with Dy^3+^ ions of greater ionic radii (0.99 Å). The observed increase in *a*_exp_ indicated the substitution of Dy^3+^ ions into Co_0.4_Cu_0.1_Zn_0.5_Fe_2_O_4_ possibly inducing tensile strain into the lattice by deforming it elastically.

**Fig. 3:**
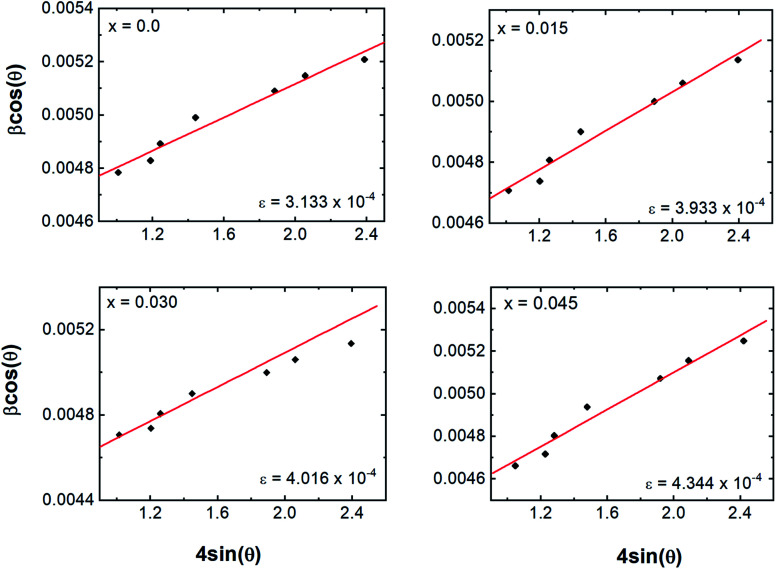
Williamson–Hall analysis plots of *β* cos *θ versus* 4 sin *θ* of Co_0.4_Cu_0.1_Zn_0.5_Dy_*x*_Fe_2−*x*_O_4_.

The nanocrystalline nature of the samples obtained from the Debye–Scherrer relationship and the microstrain induced in the crystal lattice were studied by using the Williamson–Hall (W–H) relationship^22^1
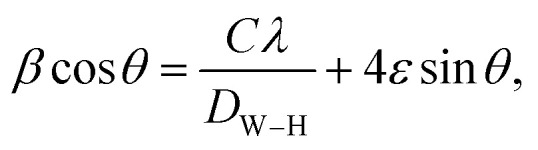
where *C* = 0.94 for the nanocrystals of uniform size, *D*_W–H_ is the crystallite size, *λ* is incident wavelength, and *ε* is the microstrain induced in the crystal lattice. In comparison to the Debye–Scherrer relationship, W–H analysis gives better information about crystallite size.^23^ Values of *ε* and *D*_W–H_ were obtained from the slope and *y*-intercept of the fitted line drawn between 4 sin *θ* and *β* cos *θ* as shown in Fig. 3. Strain values are given in the inset of Fig. 3 and *D*_W–H_ values for various Dy concentrations are shown in Fig. 2b. *D*_W–H_ obtained from W–H plots were observed to range from 31.76 to 34.40 nm for the tested concentrations, and the strain values ranged from 3.133 × 10^−4^ to 4.344 × 10^−4^. Positive values of slope here indicated that the tensile type of strain induced on the lattice increased with the Dy substitution. The increase in tensile strain with Dy substitution was also consistent with the observed change in the lattice length described above.

The crystallite diameter *D*_XRD_ of the powders was also obtained by using the peak broadening (*β*) and Bragg angle (*θ*) of the (311) peak from the well-known Debye–Scherrer^23^ equation *D*_XRD_ = (*Cλ*/*β* cos *θ*), where *C* = 0.94 and *λ* = 1.5406 × 10^−10^ m. Addition of Dy^3+^ ions into Co–Cu–Zn ferrites increased the *D*_XRD_ from 39.57 to 41.31 nm (Fig. 2b). The specific surface area (*S*) was computed by using the relationship^24^2
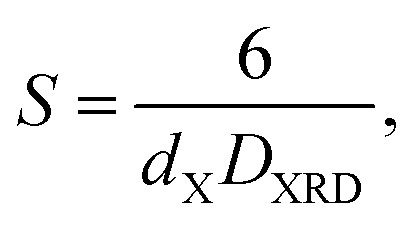
where *d*_X_ is X-ray density and 6 is the shape factor. As shown in Fig. 2a, *S* decreased from 39.37 m^2^ g^−1^ to 34.47 m^2^ g^−1^ as Dy was substituted. This decrease was related to the increased *D*_XRD_ of the samples with Dy substitution.

### Cation distribution

The Bertaut method^25^ was adopted to compute the cation distribution in Co_0.4_Cu_0.1_Zn_0.5_Dy_*x*_Fe_2−*x*_O_4_. When using this method, a few pairs of the most sensitive reflections were selected according to the expression (*I*^Obs.^_*hkl*_/*I*^Obs.^_*h*′*k*′*l*′_) = (*I*^Cal.^_*hkl*_/*I*^Cal.^_*h*′*k*′*l*′_), where *I*^Cal.^_*hkl*_ and *I*^Obs.^_*hkl*_ denote the calculated and observed intensities, respectively. Cation distribution was obtained by comparing the ratios of calculated and experimental intensities for the (220), (400) and (440) reflections. These reflections have been shown (i) to be not dependent on oxygen, (ii) to have similar intensities, (iii) to have intensities varying inversely with cation occupancy. The corresponding integrated intensity *I*_*hkl*_ was evaluated by using the relationship derived by Buerger,^26,27^ namely

3 *I*_*hkl*_ = |*F*|_*hkl*_^2^*PL*_P_,

where *L*_P_, *P* and *F* denote the Lorentz polarization, multiplicity and structure factor, respectively. *L*_P_ was obtained by using the relationship^23^4
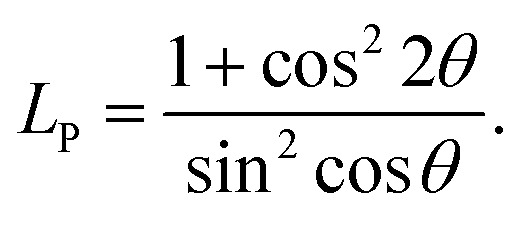


The cation distribution of Co_0.4_Cu_0.1_Zn_0.5_Dy_*x*_Fe_2−*x*_O_4_ spinel ferrite system obtained using the XRD technique is depicted in Table 1. Cu^2+^ ions occupied tetrahedral A site and octahedral B site. Zn^2+^ ions showed a preference for the A site whereas Co^2+^ ions occupied the B site. Dy^3+^ occupied the octahedral B site by replacing Fe^3+^ from the octahedral sites, a result consistent with the high B-site energy of RE^3+^ ions.

**Table 1:** Distributions of cations at the tetrahedral A site and octahedral B site and intensity ratios for Co_0.4_Cu_0.1_Zn_0.5_Dy_*x*_Fe_2−*x*_O_4_

Comp. *x*	Cation distribution		Intensity ratios			
			(220/440)		(440/422)	
	A site	B site	Obs.	Cal.	Obs.	Cal.
0.0	Cu_0.05_Zn_0.5_Fe_0.45_	Cu_0.05_Co_0.4_Fe_1.55_	0.692	0.701	3.957	4.094
0.015	Cu_0.05_Zn_0.5_Dy_0.005_Fe_0.445_	Cu_0.05_Co_0.4_Dy_0.01_Fe_1.54_	0.709	0.714	4.056	4.073
0.03	Cu_0.05_Zn_0.5_Dy_0.01_Fe_0.44_	Cu_0.05_Co_0.4_Dy_0.02_Fe_1.53_	0.708	0.729	4.069	4.043
0.045	Cu_0.05_Zn_0.5_Dy_0.015_Fe_0.435_	Cu_0.05_Co_0.4_Dy_0.03_Fe_1.52_	0.716	0.736	4.056	4.024

**Fig. 4:**
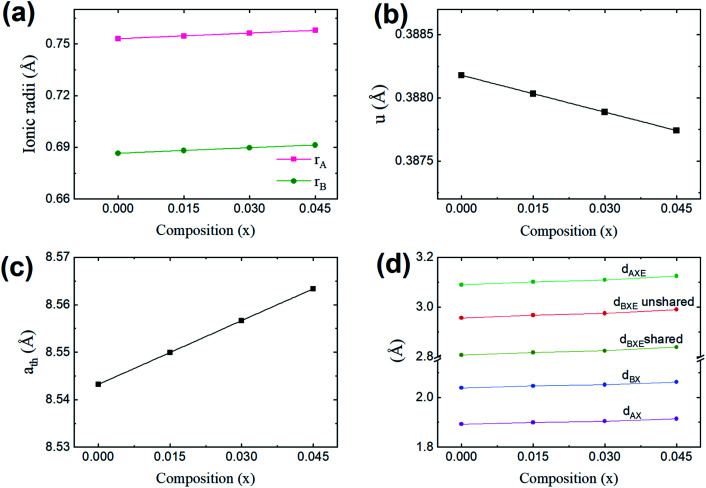
(a) Values of the mean ionic radii at the tetrahedral A site (*r*_A_) and octahedral B site (*r*_B_), (b) oxygen positional parameter (*u*), (c) theoretical lattice length (*a*_th_) and (d) allied parameters such as; tetrahedral bond (*d*_AX_), octahedral bond (*d*_BX_), tetrahedral edge (*d*_AXE_), and octahedral edge (*d*_BXE_) shared and unshared, for the Co_0.4_Cu_0.1_Zn_0.5_Dy_*x*_Fe_2−*x*_O_4_ ferrites.

Cation distribution data of Co_0.4_Cu_0.1_Zn_0.5_Dy_*x*_Fe_2−*x*_O_4_ and ionic radii of constituent ions therein were used to determine values for the ionic radius of the A site, abbreviated as *r*_A_, and that of the B site, *i.e.*, *r*_B_. As shown in Fig. 4a, *r*_A_ and *r*_B_ both increased with the Dy^3+^ substitution, attributed to the larger ionic radii of Dy ions compared to those of the Fe ions. The oxygen parameter *u* was obtained from the oxygen ion radius *R*_O_ (1.32 Å) and *r*_A_ using the equation:^27^5
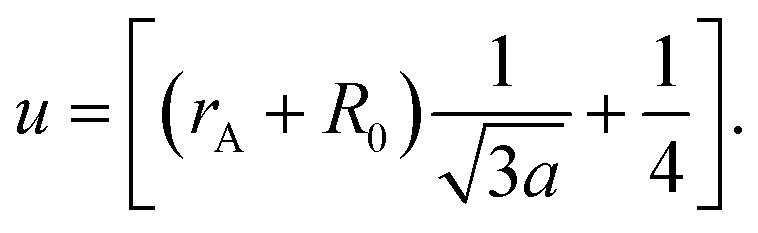


As shown in Fig. 4b, *u* decreased from 0.3882 Å to 0.3877 Å with the Dy substitution. According to many researchers, *u* would be expected to be 0.250 Å for the origin at the octahedral sites, and about 0.375 Å for the origin at tetrahedral sites and a centric crystal structure.^28,29^ According to XRD studies of some researchers, *u* > 0.375 Å for substituted ferrites.^29,30^ In the present study, *u* was determined to be higher than its expected “ideal” value of 0.375 Å.

The theoretical lattice length, *a*_th_, was determined using the relationship^31^6
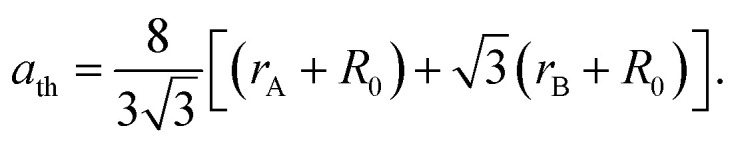


As shown in Fig. 4c, *a*_th_ increased from 8.543 to 8.563 Å with the Dy^3+^ substitution in Co_0.4_Cu_0.1_Zn_0.5_Fe_2_O_4_ ferrite.

Shared and unshared octahedral edges (*d*_BXE_ and *d*_BXEU_), the tetrahedral edge (*d*_AXE_) and tetrahedral and octahedral bond lengths (*d*_AX_ and *d*_BX_) were obtained using relationships discussed elsewhere. As shown in Fig. 4d, *d*_BXE_, *d*_BXEU_, *d*_AX_, *d*_BX_ and *d*_AXE_ increased with the Dy^3+^ substitution. Such a change was ascribed to the increase in the value of *a*_exp_ of Co_0.4_Cu_0.1_Zn_0.5_Fe_2_O_4_ with Dy^3+^ substitution.

### Transmission electron microscopy

**Fig. 5:**
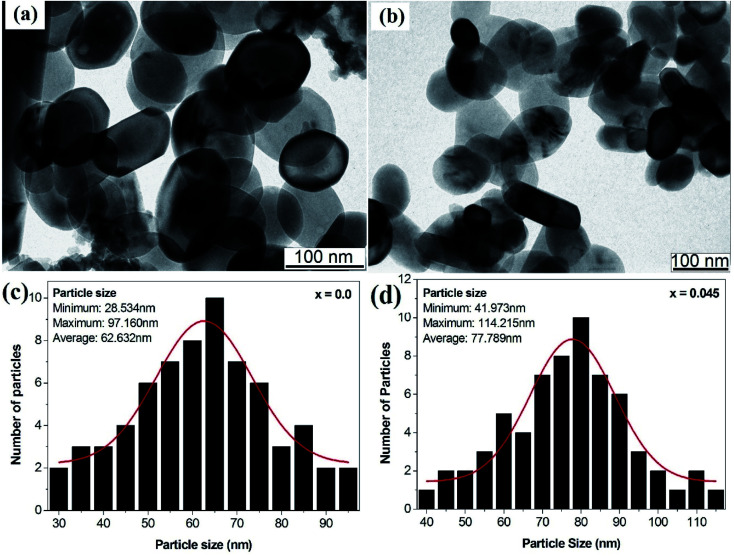
(a) TEM image of the *x* = 0.0 sample, (b) TEM image of the *x* = 0.045 sample, and (c) histogram of the particle size distribution for the *x* = 0.0 sample and (d) histogram of the particle size distribution for the *x* = 0.045 sample of the Co_0.4_Cu_0.1_Zn_0.5_Dy_*x*_Fe_2−*x*_O_4_ ferrites.

Fig. 5a and b depict the transmission electron microscopy images of the *x* = 0.0 and *x* = 0.045 samples, respectively. TEM images revealed the homogeneity of the sol–gel-synthesized samples with uniform particle size distribution. Most of the particles showed spherical shapes, in accordance with the tendency of cubic crystals to form spherical shapes in order to minimize surface tension.^32,33^ The agglomerations observed in the particles may be related to the interactions of magnetic dipoles arising within the ferrite nanoparticles.^34^ The main purpose of our acquiring the TEM images was to estimate the particle size, which was measured by employing *ImageJ* software (Version 1.52V). Fig. 5c and d show the histograms of the particle size distribution for the *x* = 0.0 and *x* = 0.45 samples, respectively. For the *x* = 0.0 sample, the particle sizes were observed to be in the range 28.53 to 97.16 nm with an average value of 62.632 nm. For the *x* = 0.045 sample, the particle sizes were observed to be in the range 41.97 to 114.215 nm with an average value of 77.789 nm.

### Elastic properties

**Fig. 6:**
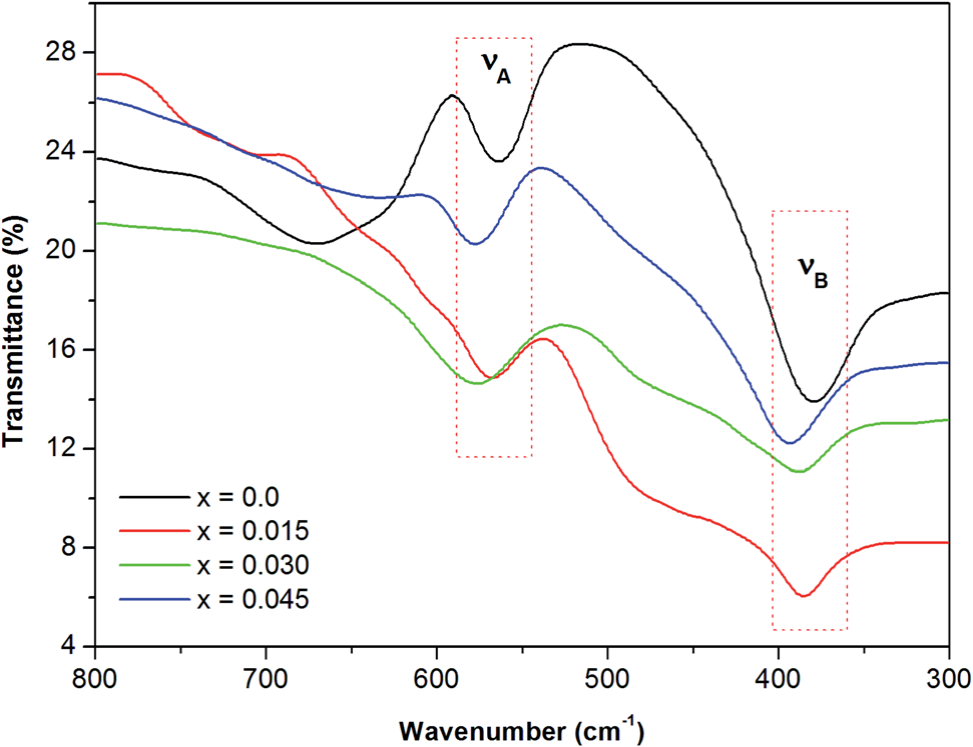
Infrared spectra of all of the investigated samples of the Co_0.4_Cu_0.1_Zn_0.5_Dy_*x*_Fe_2−*x*_O_4_ ferrites.

Infrared spectra spanning wavenumbers of 300–800 cm^−1^ were obtained for the Co_0.4_Cu_0.1_Zn_0.5_Dy_*x*_Fe_2−*x*_O_4_ samples (Fig. 6). The vibration bands observed in the vicinity of *ν*_A_ ∼ 585 cm^−1^ and *ν*_B_ ∼ 375 cm^−1^ were related to the stretching vibrations of metal ion and oxygen bond at the A and B sites, respectively, which confirmed that the powders crystallized in the cubic spinel structure.^35^

For the present series, *ν*_A_ and *ν*_B_ varied with the Dy^3+^ ions substitution in Co_0.4_Cu_0.1_Zn_0.5_Fe_2_O_4_ (Table 2). The insertion of Dy^3+^ ions was seen to shift *ν*_A_ and *ν*_B_ slightly towards higher frequencies. This shifting of absorption bands was ascribed to the crystallite size^35^ and cation occupancy. The force constant of the A site, denoted as *K*_T_, and that of the B site, denoted as *K*_O_, were estimated by employing relationships discussed elsewhere.^36^*K*_T_ and *K*_O_ were computed by using the positions of the high- and low-frequency bands *ν*_A_ and *ν*_B_ and are presented in Table 2. The average force constant was obtained using the expression *K*_av_ = (*K*_O_ + *K*_T_)/2, and the values are given in Table 2.

**Table 2:** Band positions (*ν*_A_ and *ν*_B_), force constants (*K*_T_, *K*_O_ and *K*_av_), elastic parameters such as stiffness constant (*C*_11_), longitudinal wave velocity (*V*_l_), transverse wave velocity (*V*_t_), bulk modulus (*B*), rigidity modulus (*G*), Poisson's ratio (*σ*), Young's modulus (*E*), mean wave velocity (*V*_m_) and Debye temperatures (*θ*_D_) obtained using the Waldron and Anderson methods for Co_0.4_Cu_0.1_Zn_0.5_Dy_*x*_Fe_2−*x*_O_4_

Comp. ‘*x*’	0.0	0.015	0.030	0.045
*ν*_A_ (cm^−1^)	564.19	567.08	575.76	577.69
*ν*_B_ (cm^−1^)	379.02	384.81	387.70	393.49
*K*_T_ (×10^5^ dynes per cm)	195.49	198.81	206.29	209.03
*K*_O_ (×10^5^ dynes per cm)	120.30	124.84	127.58	132.29
*K*_av_ (×10^5^ dynes per cm^−1^)	157.90	161.82	166.93	170.66
*C*_11_ (GPa)	189.32	192.30	196.32	199.72
*V*_l_ (m s^−1^)	5889.60	5996.52	6133.83	6211.11
*V*_t_ (m s^−1^)	3400.36	3462.09	3541.37	3585.99
*B* (GPa)	189.32	192.30	196.32	199.72
*G* (GPa)	63.11	64.10	65.44	66.57
*σ*	0.35	0.35	0.35	0.35
*E* (GPa)	170.39	173.07	176.69	179.75
*V*_m_ (m s^−1^)	3775.04	3843.58	3931.59	3981.13
*Θ*_D_ (K) (Waldron)	678.17	684.41	692.73	698.28
*θ*_D_ (K) (Anderson)	539.17	544.03	550.72	554.97

Force constants obtained from infrared spectra and the crystallographic parameters were used to calculate the stiffness constant and various elastic parameters. According to Waldron,^35^ stiffness constant *C*_11_ = *C*_12_ for the materials possessing the cubic spinel crystal structure and is given by the equation *C*_11_ = *K*_av_/*a*. Various elasticity parameters such as rigidity modulus *G*, bulk modulus *B*, Young's modulus *E*, longitudinal wave velocity *V*_l_, transverse wave velocity *V*_t_, mean wave velocity *V*_m_ and Poisson's ratio *σ* were calculated by using the relationships^37^7
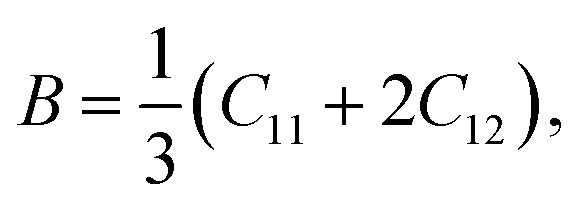

8 *V*_l_ = (*C*_11_/*ρ*)^1/2^,

9 *V*_t_ = *V*_l_/3,

10 *G* = *ρV*_t_2,

11
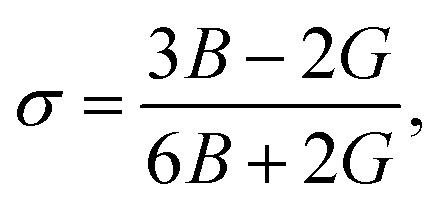

12 *E* = (1 + *σ*)2*G*,

and13
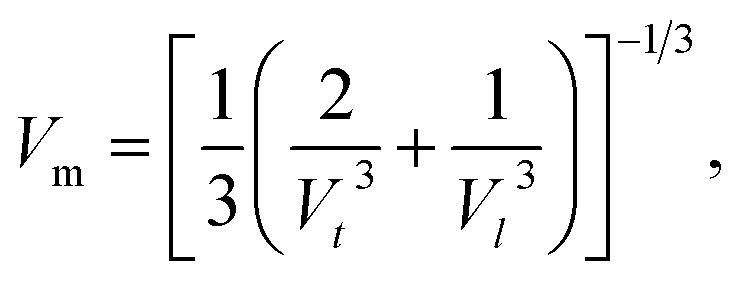


The changes in the various elasticity parameters and modulus with Dy^3+^ being substituted into the Co–Cu–Zn ferrite system are presented in Table 2. Stiffness constant *C*_11_ and all elastic moduli (*B*, *G*, and *E*) increased with the addition of Dy^3+^ ions into the Co–Cu–Zn ferrites. Poisson's constant was indeed constant (*σ* = 0.35) for all of the investigated compositions. According to isotropic elasticity theory^35,38,39^ and literature reports, Poisson's ratio must be in the range −1 to 0.5 in order to achieve good elastic behaviour. In general, elastic moduli values are related to the interionic bonding. As suggested by A. Bhaskar,^40^ strong bonding between the ions would enhance the values of the elastic moduli. In the present case, the increasing values of the elastic moduli with the addition of Dy^3+^ ions could ascribed to the increasing interatomic bonding between the ions of the Dy^3+^-substituted Co–Cu–Zn ferrites.

Debye temperatures (*θ*_D_) values of all of the investigated compositions were estimated from the relationship suggested by Anderson,^41^ namely14
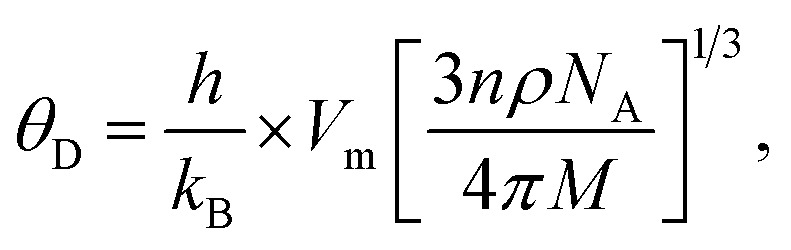
where *k* and *h* are Boltzmann's and Planck's constants, respectively, *N*_A_ is Avogadro's number, *n* is the number of atoms per unit cell (for cubic spinel, *n* = 8), *ρ* denotes density, *V*_m_ is the mean wave velocity, and *M* is the molecular weight. The Debye temperature can also be obtained by using the relationship suggested by Waldron,^35^ namely15
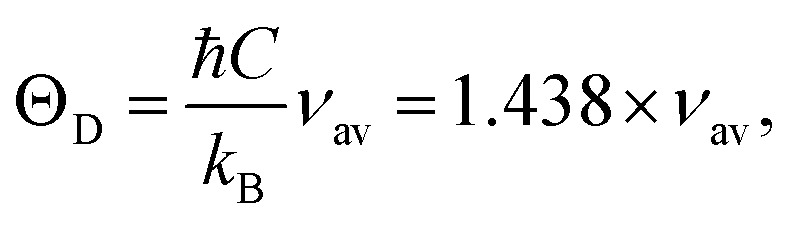
where *ν*_av_ is the average of the absorption bands (*ν*_A_, *ν*_B_) and is given by *ν*_av_ = (*ν*_A_ + *ν*_B_)/2. Both values of Debye temperatures obtained from the relationships suggested by Anderson and Waldron are given in Table 2. Addition of Dy^3+^ ions increased *θ*_D_ from 539.17 to 554.97 K and *Θ*_D_ from 678.17 to 698.28 K. The change in Debye temperature may be understood based on the theory of specific heat.^42^ Increasing P-type conduction in the materials may have increased the Debye temperature. Also, the increased rigidity of the Dy^3+^-doped Co–Cu–Zn ferrites may have contributed to its increased Debye temperature.

The elastic moduli (*B*, *G* and *E*) are not reliable unless the correction made to the zero porosity, which can be obtained by the void fraction (*P*) using the Hosselmann and Fulrath formula^40^16
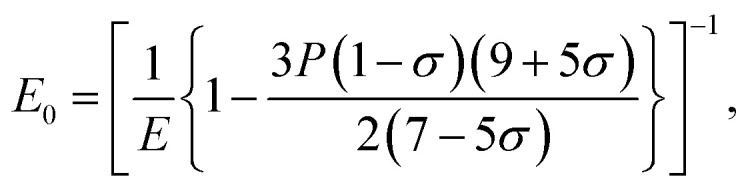
17
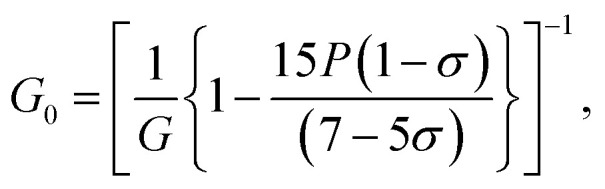
18
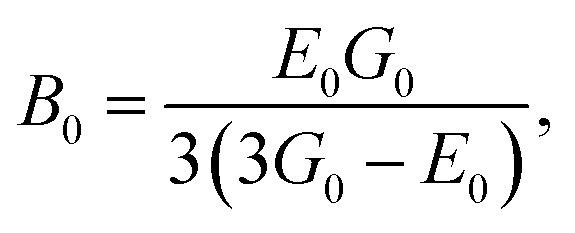
and19
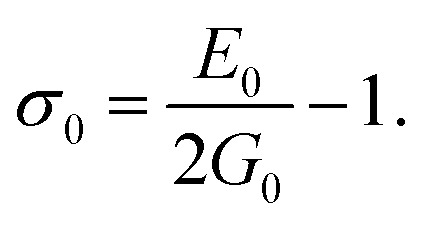


Void-free corrected values of Poisson's ratio (*σ*_0_), rigidity modulus (*G*_0_), Young's modulus (*E*_0_) and bulk modulus (*B*_0_) are presented in Table 3. All of these elastic moduli of Co–Cu–Zn ferrites tended to increase with the increasing amount of substituted Dy^3+^ ions, consistent with above-described corresponding strengthening of the bonding between the corresponding ions.

**Table 3:** Void-free corrected values of elastic parameters, including Poisson's ratio (*σ*_0_), rigidity modulus (*G*_0_), Young's modulus (*E*_0_) and bulk modulus (*B*_0_), for Co_0.4_Cu_0.1_Zn_0.5_Dy_*x*_Fe_2−*x*_O_4_

Comp. ‘*x*’	0.0	0.015	0.030	0.045
*E*_0_ (GPa)	198.07	203.55	210.27	216.48
*G*_0_ (GPa)	72.54	74.47	76.86	79.05
*B*_0_ (GPa)	254.08	254.29	265.30	275.95
*σ*_0_	0.365	0.367	0.368	0.369

### Magnetic properties

**Fig. 7:**
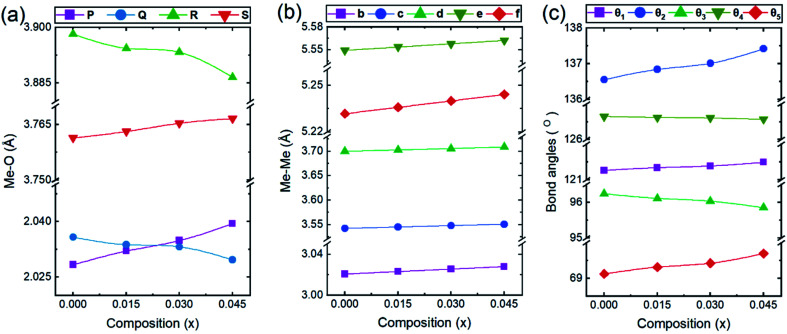
The calculated values of (a and b) the interionic distances for the (a) cation–anion Me^I^–O (*p*, *q*, *r*, and *s*), (b) cation–cation Me^I^–Me^II^ (*b*, *c*, *d*, *e*, and *f*), and (c) bond angles (*θ*_1_, *θ*_2_, *θ*_3_, *θ*_4_, and *θ*_5_).

**Fig. 8:**
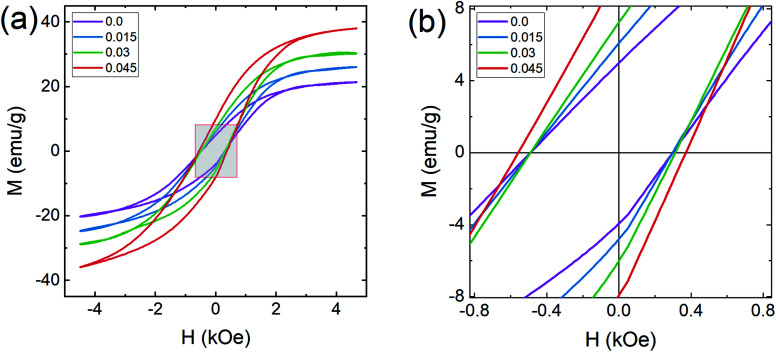
Magnetization curves of Co_0.4_Cu_0.1_Zn_0.5_Dy_*x*_Fe_2−*x*_O_4_. (a) Magnetization (*M*) as a function of applied magnetic field (*H*) and (b) expanded view of the *M*–*H* plots at low applied magnetic field strengths.

In general, three magnetic interactions such as A–A, A–B and B–B take place between the cations and neighboring anions in spinel ferrites through a super-exchange mechanism. The strengths of the energies of interaction between the magnetic ions (Me^I^ and Me^II^) are related to the cation–anion bond lengths and the Me^I^–O–Me^II^ angle (*θ*). Apparently, in general, the length of the A–B bond is less than that of the A–A bond. Thus, an A–B super-exchange interaction would be stronger than A–A and B–B super-exchange interactions. The maximum angle *θ* of 180° connecting the cations would result in the highest interaction energy. The cation–anion (Me^I^–O) (*p*, *q*, *r*, and *s*) and cation–cation (Me^I^–Me^II^) (*b*, *c*, *d*, *e*, and *f*) interionic distances, and bond angles (*θ*_1_, *θ*_2_, *θ*_3_, *θ*_4_, and *θ*_5_) for the cations and cation–anion, were determined by using the equations listed in Table 4.^43^Fig. 7a–c show that the cation distances, Me^I^–Me^II^, increased with an increasing amount of Dy substituted in the Co–Cu–Zn ferrite. In the case of distances between the cation and anion, namely Me^I^–O, P and S increased whereas *Q* and *R* decreased with an increasing amount of Dy substituted. The substitution of larger Dy^3+^ ions for Fe^3+^ ions at the BO_6_ octahedral site resulted in an increased bond length. As shown Fig. 7c, the angles associated with the A–B interaction (*θ*_1_ and *θ*_2_) increased, those associated with the B–B interaction (*θ*_3_ and *θ*_4_) decreased, and that associated with the A–A interaction (*θ*_5_) increased by small margin with an increasing amount of Dy^3+^ substituted. The variation in bond angles revealed that A–O–B interaction, which governs the magnetization in the system, strengthened with an increasing amount of Dy ions substituted. Such a change should increase the magnetization levels of the Co–Cu–Zn ferrites. Thus magnetic properties were investigated by employing VSM with a peak field strength of 5 kOe. Fig. 8a presents the magnetic hysteresis loops obtained from our system; these loops clearly indicated that the saturation magnetization (*M*_s_) increased with increases in Dy^3+^ concentration. The RE-Dy^3+^ ions showed a greater magnetic moment (10.48 *μ*_B_) compared to that of Fe^3+^ (5 *μ*_B_). The increased content of Dy^3+^ ions at the B sites increased the magnetization of these sites, which resulted in the increase in the *M*_s_.

**Table 4:** Expressions for determining the interionic distances for the cation–cation (Me^I^–Me^II^ (*b*, *c*, *d*, *e*, and *f*), cation–anion Me^I^–O (*p*, *q*, *r*, and *s*), and bond angles (*θ*_1_, *θ*_2_, *θ*_3_, *θ*_4_, and *θ*_5_)

Me^I^–Me^II^	Me^I^–O	Bond angles
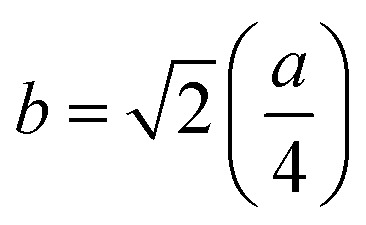	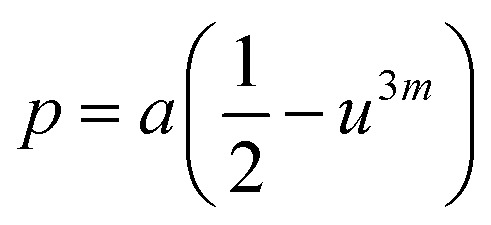	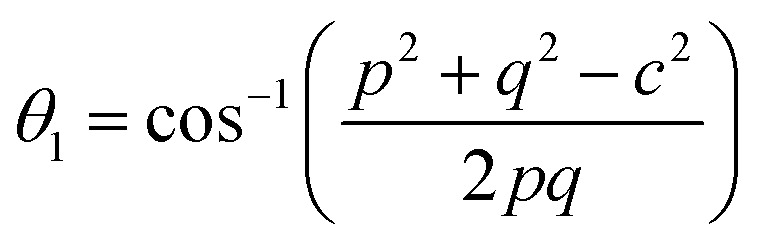
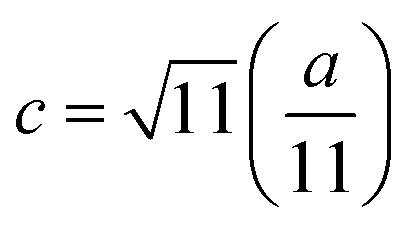	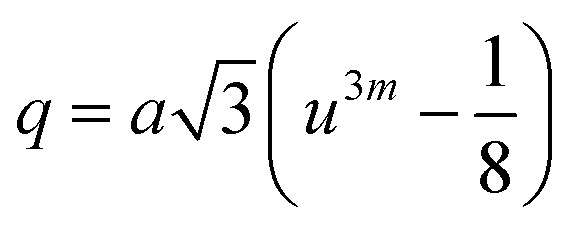	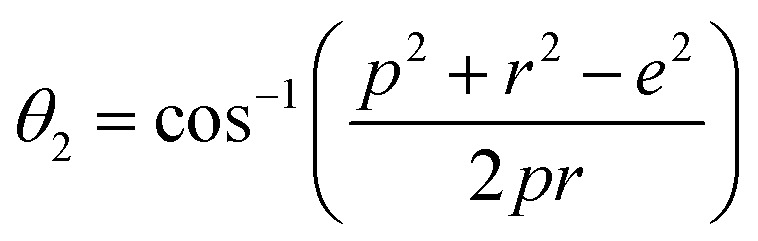
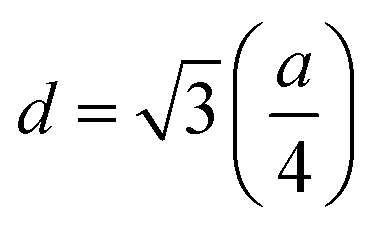	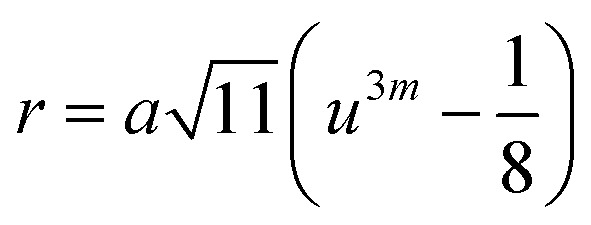	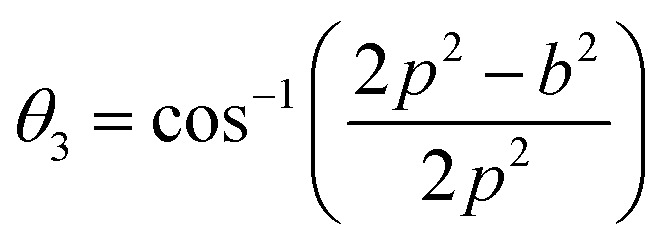
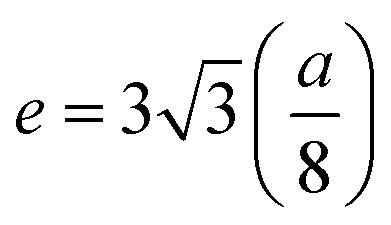	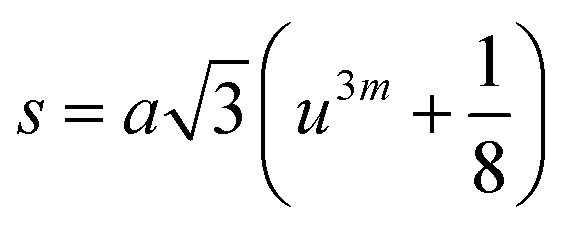	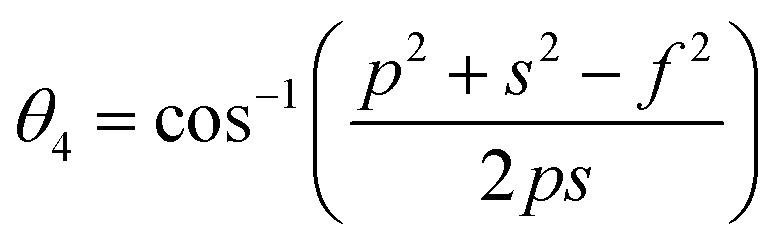
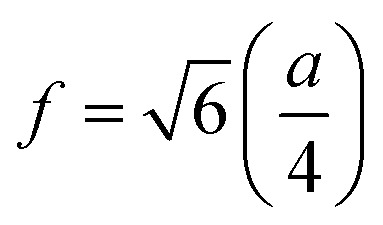		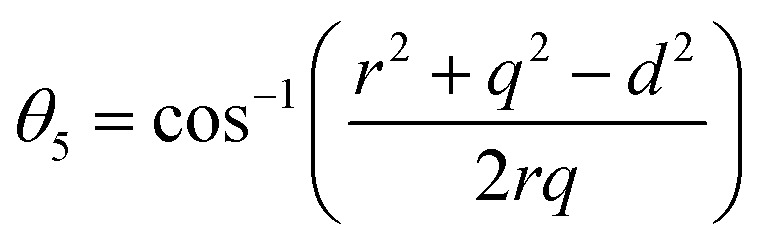

Coercivity (*H*_c_) is generally governed by the magneto-crystalline anisotropy constant (*K*_1_), permeability (*μ*_O_) and *M*_s_ through the Brown relationship^44^20
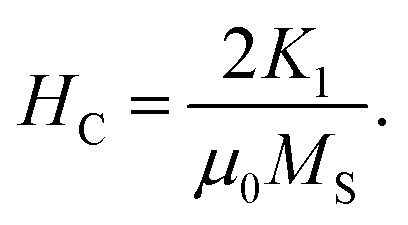


**Fig. 9:**
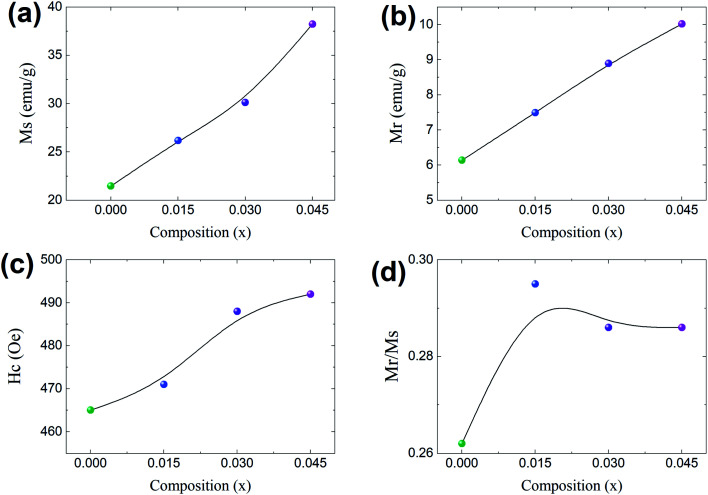
Co_0.4_Cu_0.1_Zn_0.5_Dy_*x*_Fe_2−*x*_O_4_ magnetism properties each as a function of Dy composition (*x*). (a) Magnetization (*M*_s_), (b) remnant magnetization (*M*_r_), (c) coercivity (*H*_c_), and (d) the remnant ratio *M*_r_/*M*_s_.

The coercivity value is independent of *M*_s_ and can be controlled by heat treatment or structural deformation. Values of *M*_s_, *H*_c_, remnant magnetization (*M*_r_) and remanence ratio (*R* = *M*_r_/*M*_s_), each as a function of the relative amount of Dy^3+^ substituted, are shown in Fig. 9a–d. As shown in Fig. 8b and 9c, *H*_c_ increased with increasing amount Dy^3+^ substituted. An increased *H*_c_ is in general directly related to the higher *K*_1_ of rare earth ions such as Dy.

In general, cation distribution, particle sizes and magnetic moments of the cations are the major parameters affecting magnetic properties. However, oxygen vacancies and variation in the valence state of Fe, namely between Fe^3+^ and Fe^2+^, can also influence the magnetic properties.^10^ However, in the present case, all of the materials were sintered in an air atmosphere and thus the possibility of having oxygen vacancies can be neglected. Secondly, the Dy and Fe ions in the materials used had the same valence state of 3+. Considering these factors, a change in the valence state between Fe^3+^ and Fe^2+^ can be ignored in the present work, though it can be studied in the future using XPS.

### Dielectric measurements

**Fig. 10:**
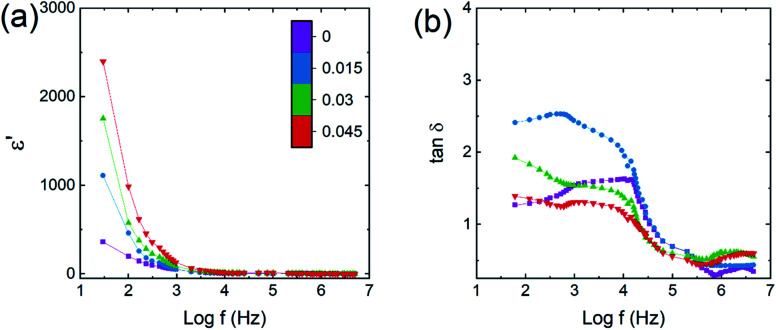
Co_0.4_Cu_0.1_Zn_0.5_Dy_*x*_Fe_2−*x*_O_4_ dielectric parameters, each as a function of logarithm of frequency (*f*). (a) Real part of dielectric constant (*ε*′) and (b) dielectric loss tangent (tan *δ*).

The dielectric constant (*ε*′) was obtained using a cylindrical ferrite pellet sample and the relationship21
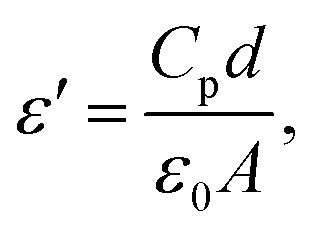
where *d* is the pellet thickness, *C*_p_ is the capacitance, *A* is the area of pellet surface and *ε*_0_ is the permittivity of free space. Fig. 10a and b show dielectric constant (*ε*′) and dielectric loss tangent (tan *δ*) values, each as a function of frequency. These two values decreased with increasing frequency, confirming the dispersion at lower frequency.^45^ The dispersion and higher value of *ε*′ at low frequency may be ascribed to the grain heterogeneity of material.^46^ The change in dielectric dispersion can be understood *via* the Maxwell–Wagner model^47,48^ and Koop's^49^ phenomenological theory. The exchange of 3d orbital electrons between Fe^2+^ and Fe^3+^ can be localized at the metal ions, resulting in a local shift of electrons (e^−^) toward the direction of the applied electric field and helping to determine the polarization strength. The variation in *ε*′ and tan *δ* at low frequency may also be related to the ‘grain-boundary-induced high resistivity’. Thus more energy is needed at the low-frequency region for the exchange of electrons between Fe^2+^ and Fe^3+^ ions, which gives higher values of *ε*′ and tan *δ*. In contrast, at the higher-frequency region, dispersion is related to ‘grain-induced lower resistivity’. Thus, less energy is needed for inter-cation electron exchange. Furthermore, a higher tan *δ* at lower frequency may be related to crystal defects, impurities and moisture. The conduction of Cu^2+^, Co^2+^, Zn^2+^ with Fe^3+^ ions at the B site is possible through following mechanisms:

22 Fe^3+^ + Cu^2+^ ↔ Fe^2+^ + Cu^3+^

23 Fe^3+^ + Zn^2+^ ↔ Fe^2+^ + Zn^3+^

24 Fe^3+^ + Co^2+^ ↔ Fe^2+^ + Co^3+^

The value of tan *δ* reflects the energy loss within a material and it appears whenever polarization does not follow the applied alternating field. A broad relaxation-related hump in each of the tan *δ* plots was observed (Fig. 10b) and can be understood based on the Rezlescu model.^50^ The condition for a broad hump in the tan *δ* of a dielectric material is related to the equation

25 *ωτ* = 1,

where *τ* is the relaxation time and *ω* = 2π*f*_max_. The value of *τ* is related to the jumping probability according to the relationship26
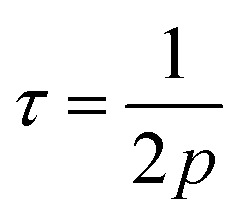
or

27 *f*_max_ ∝ *р*

Thus, a broad hump or maximum can be obtained as a result of frequency of charge hopping between cations perfectly coinciding with the frequency of the electric field. The Co–Cu–Zn ferrite samples with *x* = 0.045 showed the highest *ε*′ and lowest tan *δ*.

The increase in *ε*′ for Co–Cu–Zn ferrite with increasing amount of Dy^3+^ substituted could be mainly related to the increase in grain size and decrease in conductivity. An increase in grain size would increase the probability of hopping occurring between Fe, Co and Cu ions, resulting in an enhancement in dielectric constant. On the other hand, an increase in grain size would decrease the resistivity of the materials because of the resulting fewer grain boundaries, and that would also be beneficial for enhancing the dielectric constant and decreasing the dielectric loss tangent.

## Conclusions

Single-phase cubic spinel structured Co_0.4_Cu_0.1_Zn_0.5_Dy_*x*_Fe_2−*x*_O_4_ nanoparticles were successfully prepared using the sol–gel autocombustion route. The Dy substitution resulted in an increase in tensile strain in the materials from 3.1 (*x* = 0.0) to 4.3 × 10^−4^ (*x* = 0.045), consistent with the lattice length having increased from 8.340 to 8.545 Å. The average crystallite size increased from 39.5 to 41 nm with the Dy substitution. Zn^2+^ ions occupied the A site only; Co^2+^ and Dy^3+^ occupied the B site whereas Cu^2+^ and Fe^3+^ occupied both crystallographic sites. Force constants, elastic parameters and Debye temperatures increased with Dy substitution. Increases in grain size, magnetocrystalline anisotropy, and 4f–3d couplings and strengthening of the A–B interaction with the Dy substitution resulted in increases in all of the magnetic properties. Saturation magnetization increased from 21 to 38 emu g^−1^ whereas coercivity increased from 480 to 560 Oe with the increase in Dy substitution. The enhancement in saturation magnetization was also related to the higher magnetic moment of Dy ions compared to that of Fe ions_._ The dielectric constant increased from 355 to 2400, and dielectric loss also slightly decreased, when Dy was included. This ability to modify the magnetic properties of the prepared material makes it a suitable candidate for magnetic recording media.

## Conflicts of interest

All the authors declare no conflict of interest.

## Supplementary Material
